# The Bureau of Animal Industry

**Published:** 1884-07

**Authors:** 


### EDITORIAL DEPARTMENT.

### THE BUREAU OF ANIMAL INDUSTRY.

At last we have a tangible idea presented for Congress to discuss, with reference to the study and suppression of animal diseases in the United States.

From the tenor of the discussion which we have thus far read, it would seem that our Congressmen are no better able to deal with this subject than they are with any other, save how they can so divide our surplus revenue as to get a slice of the pickings. Even among Congressmen, there are exceptions to prevailing rules or customs.

"A Bureau of Animal Industry."

The name itself marks a step in advance of all previous attempts, indicating an undisputed fact that there is much in a name. Heretofore we have had "Cattle Commissions," but it is also a fact that they have only been such in name. They have not been "cattle commissions," but very restricted pleuro-pneumonia commissions. Any work which they have thus far done will bear no comparison with the work that single individuals have done under the auspices of the Agricultural Department.

In selecting men for the Bureau of Animal Industry, Congress should learn something by past experiences, and take new men; for the members of the existing Treasury Commission have been fully tried, and found lamentably wanting. We allude to the Foot and Mouth Disease episode at Portland, Me.

It has been surprising to every graduated man in the veterinary profession that our Government should place so low an estimation on education as to appoint an empiric in any such position; but his retention has been still more surprising after every act of his while on the Commission has proven his utter unfitness for such a position. How any graduated man with the reputation enjoyed by the only veterinarian on this Commission, could assent to such an appointment, and again consent to work in such a position with an empiric, is a still greater conundrum. The authorities that may have the duty of nominating a new commission, should make a clean sweep, and have entirely new men for the work. Any man that has any political affiliations should be condemned where technical work is concerned.

Many points in connection with these questions will be found touched upon in the articles on trichiniasis, and in the address read before the Massachusetts Veterinary Association, in this number of the JOURNAL, and so need not be alluded to here.

As said, the name is right. It indicates the work of such a bureau to be of the most comprehensive character, so that nearly every person composing it will have to be, in a measure, a specialist in a certain direction. There will have to be a strict division of labor in order to obtain any unity of result. Let us indicate what this work should be, though we fall far short of noticing it all.

First, there is the quarantining of imported cattle, which should be one in fact, not in fancy, as it is now. Each quarantine should be a Government station. Each one should have its deputy veterinary inspector. They should all be surrounded with a high fence, should have one point of entrance for imported cattle, and one for exit for those that have passed the quarantine period. Such cattle, or those already at such stations, should never be in contact, or cross paths that have been crossed by new arrivals. All quarantined animals are to be treated as diseased until their term expires. There should be a guard stationed at the entrance, day and night. No visitors should ever be allowed to enter, except they have business in connection with the cattle. Inspection for curiosity should be strictly forbidden. All persons entering should be treated as quarantined animals while there, and be obliged to change their outer garments, or put on overshoes and an outside suit supplied for the purpose. On no account should they be permitted to handle the cattle. Each lot should have its special stable and attendant; the latter should never be permitted in the stables of the others. All cattle cars or conveyances used to transport imported cattle from the ships to the stations should be thoroughly scrubbed, cleansed, disinfected, and whitewashed before being allowed to leave. They should be registered on entering the grounds, and again on leaving. The fact of their having been "cleansed" should be noted. This has never been done. We may not have touched all the necessary points, but we have endeavored to show what a quarantine should be.

The next question will be special work with reference to pleuro-pneumonia, which should be all stamped out before we are a year older.

But the best thing about the name Bureau is that it indicates that all other diseases of our animals are to be duly considered. We have only to mention trichiniasis, glanders, anthrax and anthracoid diseases, Southern cattle disease, rabies, and tuberculosis in cattle ; the inspection of dairies ; what diseases are mostly seen among animals slaughtered ; the gradual endeavor to have abattoirs that shall be town property in every locality; and a thing that needs looking after, "sheep scab." Canada is quietly shipping her scabby sheep away through our ports, and we are not taking a step to prevent it.

Next we have the export cattle business. Here is any amount of room for work. The Government should erect export quarantines at, or as near as possible, every steamship wharf, where cattle destined for export should be obliged to be unloaded, rested out for forty-eight hours, and be examined by its own veterinarian before being allowed to be shipped. It is as much to the benefit of the cattle interests of this country to be sure that no contagious, diseased, or sick cattle go abroad, as to know that none get in.

Then we have the transportation business, which should be so regulated that none but healthy animals are collected at the shipping centres upon the railroads. This includes the thorough cleansing of such places, and the cleansing and disinfection of all conveyances used for animal transport, no matter whether infectious diseases have been discovered among them or not.

All this cannot be done at once, but we must come to it finally. Without educated veterinarians nothing can be done. Without such work as this, without the additional support which such positions in the Government service give, it can never get the quality of men necessary for such work among the graduates of our schools. We have had an example in Kansas, where a graduate, and a well known one, from one of our best known veterinary schools, mistook something else for foot and mouth disease, and caused no end of disturbance, though every qualified veterinarian that read the newspaper descriptions, said to be this gentleman's own words, could have taken oath that it was not this disease at all.

Such mistakes are unpardonable, and do an immense amount of injury, as was evinced by the rumors that sprang up out West, until one would have thought that this pest was springing up, like country politicians, at every crossroad and post-office.

The task of a Bureau of Animal Industry is briefly stated in saying that it is to begin the work of organizing a national veterinary police system for the whole Union. A police system of this kind means laws and their executors. This question of animal hygiene comes more directly in contact with our national bugbear, State rights, than any other that Congress will ever have to deal with.

State rights cease when contagious diseases are liable to be transported any and everywhere, as do the rights of the great American freeman when he owns animals having such diseases, and desires to leave his own premises. His neighbors soon settle his rights. Shotguns would soon have to be called in aid, and life endangered, if the farmers in Maine that had foot and mouth disease among the cattle insisted on their rights to do as they pleased, and go over the public ways with them.

We have but indicated the way; let others work out the details of what will prove to be one of the hardest tasks the legislators of this country, State and National, have ever undertaken.

BILLINGS.

